# Deciphering Oxidative Stress in Cardiovascular Disease Progression: A Blueprint for Mechanistic Understanding and Therapeutic Innovation

**DOI:** 10.3390/antiox14010038

**Published:** 2024-12-31

**Authors:** Zhaoshan Zhang, Jiawei Guo

**Affiliations:** 1 Department of Vascular and Endovascular Surgery, The First Affiliated Hospital of Yangtze University, Jingzhou 434000, China; 2 Department of Pharmacology, School of Medicine, Yangtze University, Jingzhou 434023, China; 3 Department of Pharmacology, Cardiac and Cerebral Vascular Research Center, Zhongshan School of Medicine, Sun Yat-Sen University, Guangzhou 510080, China

**Keywords:** cardiovascular disease, oxidative stress, redox signaling, gene therapy, inflammation, ROS, antioxidants

## Abstract

Oxidative stress plays a pivotal role in the pathogenesis and progression of cardiovascular diseases (CVDs). This review focuses on the signaling pathways of oxidative stress during the development of CVDs, delving into the molecular regulatory networks underlying oxidative stress in various disease stages, particularly apoptosis, inflammation, fibrosis, and metabolic imbalance. By examining the dual roles of oxidative stress and the influences of sex differences on oxidative stress levels and cardiovascular disease susceptibility, this study offers a comprehensive understanding of the pathogenesis of cardiovascular diseases. The study integrates key findings from current research in three comprehensive ways. First, it outlines the major CVDs associated with oxidative stress and their respective signaling pathways, emphasizing oxidative stress’s central role in cardiovascular pathology. Second, it summarizes the cardiovascular protective effects, mechanisms of action, and animal models of various antioxidants, offering insights into future drug development. Third, it discusses the applications, advantages, limitations, and potential molecular targets of gene therapy in CVDs, providing a foundation for novel therapeutic strategies. These tables underscore the systematic and integrative nature of this study while offering a theoretical basis for precision treatment for CVDs. A major contribution of this study is the systematic review of the differential effects of oxidative stress across different stages of CVDs, in addition to the proposal of innovative, multi-level intervention strategies, which open new avenues for precision treatment of the cardiovascular system.

## 1. Introduction

Cardiovascular diseases (CVDs) are a significant global health and economic burden, highlighting the critical importance of maintaining cardiovascular health [1]. CVDs are among the leading causes of morbidity and mortality worldwide, primarily resulting in cardiovascular damage [2,3]. These diseases arise from a range of factors, including genetic predisposition, chronic conditions (e.g., hypertension and diabetes), unhealthy lifestyle habits (e.g., smoking and obesity), and environmental influences [4,5]. Oxidative stress is a key contributor to the pathogenesis of CVDs [6]. Research into its underlying mechanisms not only enhances our understanding of the disease but also reveals potential therapeutic targets, paving the way for more effective drugs and preventative strategies.

Oxidative stress refers to a pathological state characterized by the excessive production of reactive oxygen species (ROS) [7]. Due to their high reactivity, ROS can damage cellular components, including proteins, lipids, and DNA [8,9,10]. In cardiomyocytes, ROS can induce mitochondrial dysfunction, leading to energy metabolism disorders and myocardial injury [11]. Additionally, ROS play a role in atherosclerosis by promoting endothelial dysfunction and stimulating smooth muscle cell proliferation [12]. Targeting the oxidative stress pathway offers a promising therapeutic approach to mitigate these effects and slow CVD progression.

This review comprehensively examines the role of oxidative stress in CVDs, focusing on its heterogeneity across different disease types. It also emphasizes the importance of personalized medicine in CVD management, advocating for precision medicine strategies based on oxidative stress biomarkers as a theoretical foundation for clinical practice.

## 2. The Dynamic Role of Oxidative Stress in the Progression of Cardiovascular Disease

### 2.1. Molecular Mechanisms Underlying Oxidative Stress

**Figure 1:**
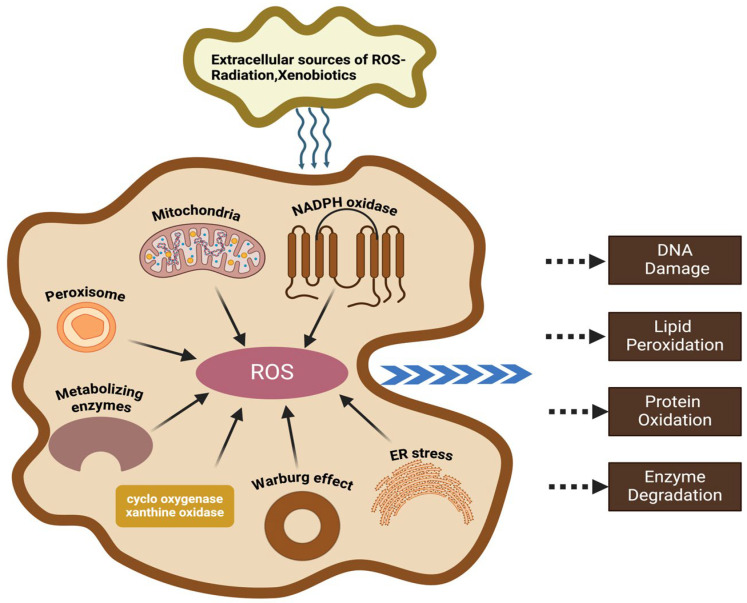
Generation and roles of ROS. ROS are highly reactive molecules containing oxygen produced during cellular metabolism. Excessive ROS production can lead to oxidative stress, resulting in cellular damage. As depicted in the figure, intracellular ROS primarily originate from the electron transport chain (ETC), NADPH oxidase (NOX), xanthine oxidase (XO), cyclooxygenase (COX), nitric oxide synthase (NOS), and cytochrome P450 (CYP450). ROS can oxidize biomolecules, including lipids, proteins, and DNA, impairing cellular structure and function, triggering inflammatory responses, and promoting apoptosis, ultimately contributing to the pathogenesis of CVDs.

Oxidative stress refers to an imbalance between the production of ROS and antioxidant defenses, leading to oxidative damage in cells and tissues (Figure 1). This imbalance is implicated in the pathogenesis of various diseases, including CVDs and neurodegenerative disorders.

ROS are pivotal in the balance between physiological signaling and pathological oxidative stress (Figure 1). The electron transport chain (ETC) in a mitochondrion serves as a primary source; electron leakage generates superoxide radicals, particularly under the conditions of impaired oxidative phosphorylation [13]. NOX enzymes are another major contributor, producing ROS as immune responses and cellular signals, but their dysregulation is linked to oxidative damage [14]. Similarly, xanthine oxidase (XO) catalyzes purine metabolism, generating superoxide and hydrogen peroxide, which are exacerbated during ischemia-reperfusion injury [15]. Endoplasmic reticulum (ER) stress further amplifies oxidative stress by disrupting protein folding and activating ROS-producing pathways [16]. Together, these systems orchestrate a delicate interplay between ROS production and cellular homeostasis, with dysregulation contributing to the pathogenesis of numerous diseases.

The ETC, which is a series of protein complexes embedded in the inner mitochondrial membrane, plays a central role in oxidative phosphorylation [17,18]. The mitochondrial respiratory chain consists of four transmembrane protein complexes that facilitate electron transfer and oxidative phosphorylation [19]. Complexes I and II introduce electrons from NADH and FADH₂ into the respiratory chain [20,21]. Complex III transfers electrons within the lipid membrane, coupling this process with proton translocation [22]. Complex IV, the terminal oxidase, transfers electrons to oxygen molecules, producing water and completing the final proton pumping process [23]. As electrons are transferred along this chain, a proton gradient is established across the inner membrane, driving ATP synthesis [24]. However, a small proportion of electrons may leak from the chain and react with molecular oxygen, generating superoxide anions—the primary ROS produced in mitochondria [19,25]. The uncoupling of oxidative phosphorylation, coupled with structural or functional abnormalities in the electron transport chain complexes, can lead to uncontrolled electron flow, increasing the risk of electron leakage and inducing oxidative damage [26,27]. Proton leak occurs in two forms: basal (associated with the mitochondrial inner membrane lipid bilayer and adenine nucleotide translocator [ANT]) and inducible (regulated by uncoupling proteins [UCPs] and ANT) [28,29]. Both constitutive and regulatory proton transporters are critical in maintaining cellular energy homeostasis. Targeting these transporters with pharmacological interventions holds promise as a novel therapeutic strategy for mitochondrial diseases.

NOX, a multi-subunit membrane-bound enzyme complex, is the primary enzyme responsible for the generation of superoxide anion (O_2_^−^) through the reduction of molecular oxygen [30]. This initial step leads to the production of a variety of ROS (Figure 1). The NOX family can be broadly categorized into two groups based on structural and functional characteristics: classical NOX isoforms (Nox1–Nox4), which typically form complexes with the p22phox subunit, and dual oxidase (Duox) isoforms (Duox1 and Duox2), which require the Duoxa subunit for their activity [31]. NOX-derived ROS synergize with growth factors such as VEGF and PDGF to promote endothelial cell proliferation, migration, and tube formation through the activation of downstream signaling pathways, thereby driving angiogenesis [32,33]. Nox2 and Nox4 form heterodimers in the heart [34]. Nox2-derived ROS play a central role in the pathogenesis of angiotensin II-induced left ventricular hypertrophy and cardiac remodeling associated with pressure overload [35]. Additionally, Nox2 is involved in the cardioprotective effects of ischemic preconditioning [36]. Cardiac Nox4 expression is upregulated under various stress conditions, leading to increased ROS production. Nox5, a unique calcium-dependent NADPH oxidase, is widely expressed in the cardiovascular system [37]. Unlike its homologs, Nox5 can be activated by calcium ions without the need for additional subunits, making it a key enzyme in ROS generation in cardiovascular diseases [38]. The overexpression of Nox5 is closely associated with various cardiovascular diseases, including hypertension, aneurysms, and atherosclerosis [39]. NOX-dependent redox signaling, characterized by the generation of localized and low levels of ROS, regulates a variety of cellular processes, including proliferation, apoptosis, and migration [40].

XO is a key oxidoreductase that initially catalyzes the oxidation of hypoxanthine to xanthine and subsequently catalyzes the oxidation of xanthine to uric acid [41]. During this process, XO uses molecular oxygen as an electron acceptor, generating O_2_^−^ and initiating a cascade of oxidative reactions that produce various ROS [15,42]. XO contains several electron transfer centers, including a molybdenum cofactor, FAD, and iron–sulfur clusters [42]. During catalysis, electrons are transferred from the substrate (hypoxanthine or xanthine) to these centers and, ultimately, to molecular oxygen. As a result, molecular oxygen is reduced to superoxide anion, which then initiates a cascade of reactions that generate various ROS, including hydrogen peroxide (H_2_O_2_) and hydroxyl radical (OH) [43,44].

ER stress, triggered by the accumulation of unfolded or misfolded proteins, can lead to apoptosis (Figure 1). Increased cytosolic Ca^2+^, released from the ER, activates kinases such as PKC and CaMKII, which phosphorylate and activate NADPH oxidases, leading to ROS generation [45,46]. The unfolded protein response (UPR) is a cellular adaptive response to ER stress that is primarily mediated by three transmembrane ER proteins: IRE1α, PERK, and ATF6 [16,47]. These sensors detect unfolded proteins in the ER lumen and initiate downstream signaling pathways. The primary mechanism underlying ROS generation during ER stress involves electron transfer and oxidative reactions during protein folding. Protein disulfide isomerase (PDI) plays a crucial role in catalyzing disulfide bond formation, and electron transfer in this process leads to the oxidation of ERO1, resulting in ROS production [48]. Nox4 interacts with PDI and directly generates superoxide anions [49,50]. Additionally, glutathione (GSH), an antioxidant, contributes to ROS production when its redox balance is disrupted [51]. The microsomal monooxygenase system, particularly cytochrome P450 2E1, also generates ROS during electron transfer [52]. Collectively, these mechanisms disrupt cellular redox homeostasis, ultimately leading to cellular damage or death.

Peroxisomes are cellular organelles responsible for various important oxidative reactions [53]. They are involved in the metabolism of fatty acids (through α-oxidation), very long chain fatty acids (VLCFAs; through β-oxidation), and purines [54]. Within peroxisomes, oxidases catalyze the oxidation of substrates, producing H_2_O_2_. To mitigate the harmful effects of hydrogen peroxide, peroxisomes contain a robust antioxidant defense system, including catalase, glutathione peroxidase, epoxide hydrolase, PMP20, and peroxidases (I, V, and VI), which collectively detoxify H_2_O_2_ [55,56]. The protective role of catalase in peroxisomes is supported by studies in human fibroblasts from patients with multiple peroxisomal enzyme deficiencies and catalase deficiency, as well as in experimental animal models, such as rats with alcoholic cardiomyopathy [57,58]. The accumulation of hydrogen peroxide in peroxisomes, resulting from either increased oxidase activity or impaired antioxidant defenses, can induce oxidative stress [59]. Therefore, understanding the interplay between peroxisomes, ROS, and oxidative stress is crucial for elucidating the cellular mechanisms underlying health and disease.

### 2.2. Sex-Based Differences in Redox Status and Their Implications for CVD

Young men are more susceptible to coronary heart disease, myocardial infarction, and other ischemic heart diseases due to their lower estrogen levels and higher inflammatory burden [60]. Postmenopausal women experience an increased risk of cardiovascular diseases as estrogen levels decline [61]. Female patients with cardiovascular diseases exhibit highly variable clinical presentations that are often characterized by atypical symptoms, resulting in delayed diagnosis [62]. Additionally, women have poorer outcomes in certain cardiovascular conditions, which may be linked to sex-based differences in inflammatory responses and microvascular function.

Sex differences in mitochondria represent a promising area of research in cardiovascular diseases. Mitochondrial diseases, often caused by mutations in either mitochondrial or nuclear DNA, can have a greater impact on males than females. Mitochondrial dysfunction can lead to a variety of diseases, including cardiomyopathy, and these diseases often exhibit sex-specific differences [63].

Sex hormones, particularly estrogen and testosterone, exert significant influences on oxidative stress levels [64]. Estrogen enhances the body’s antioxidant capacity by activating the Nrf2/Keap1 signaling pathway and upregulating the expression of antioxidant enzymes such as superoxide dismutase (SOD) and glutathione peroxidase (GPx) [65]. The regulatory effects of androgens on ROS are more complex and may depend on hormone concentration.

The activation of the Nrf2 and PI3K/Akt pathways, coupled with the preservation of mitochondrial function by estrogen, enhances the antioxidant defense and promotes cell survival in the female cardiovascular system [66]. Consequently, women exhibit greater resistance to oxidative stress compared to men, who exhibit weaker signaling in these pathways [67]. Sex differences significantly influence the development and progression of cardiovascular diseases by modulating sex hormones, antioxidant systems, and redox signaling pathways.

### 2.3. Oxidative Stress Profile at Various Stages of CVDs

**Figure 2:**
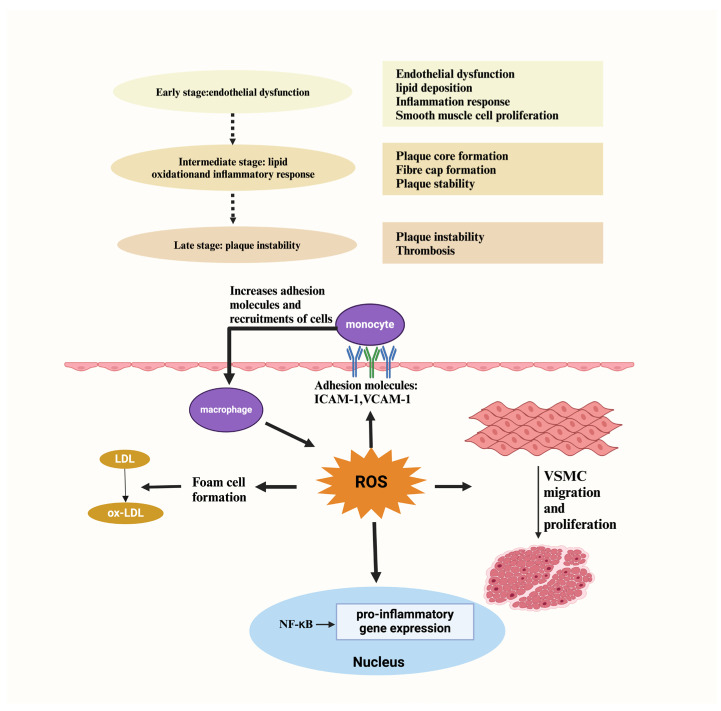
Pathophysiological processes in CVDs. This figure provides a visual representation of the characteristic features of oxidative stress at different stages of cardiovascular diseases and their potential underlying causes, demonstrating the close relationship between oxidative stress and disease progression.

Oxidative stress levels fluctuate dynamically across different CVDs, with both the magnitude and targets of oxidative stress evolving as the diseases progress [68]. The role of oxidative stress in the developmental process of cardiovascular disease is shown in Figure 2. This dynamic process creates a vicious cycle that exacerbates disease severity (Figure 2). By elucidating the temporal changes in oxidative stress during CVD progression, we can gain deeper insights into disease pathogenesis and identify novel therapeutic targets for antioxidant interventions.

#### 2.3.1. Early Triggering Mechanisms of Oxidative Stress in Endothelial Injury

As a pivotal regulator of cardiovascular homeostasis, the vascular endothelium is influenced by both genetic and epigenetic mechanisms [69]. Endothelial dysfunction, which is characterized by reduced nitric oxide bioavailability, increased adhesion molecule expression, and enhanced permeability, is a hallmark of atherosclerosis that initiates a cascade of events leading to plaque formation [70].

Elevated lipoprotein permeability, which is exacerbated oxidative stress, enhanced monocyte adhesion, abnormal extracellular matrix remodeling, and dysregulated coagulation/fibrinolysis, collectively characterizes endothelial dysfunction [71]. In the pathogenesis of atherosclerosis, various pathophysiological stimuli—such as pro-inflammatory cytokines, pathogen-associated molecular patterns (PAMPs), advanced glycation end products (AGEs), oxidized low-density lipoprotein (ox-LDL), and its components (e.g., lysophosphatidylcholine)—synergistically induce and aggravate endothelial dysfunction, thereby promoting the formation and progression of atherosclerotic plaques [72].

The formation of atherosclerotic lesions is closely linked to the pro-inflammatory activation of endothelial cells. Upon injury, endothelial cells lose their anti-inflammatory and anti-thrombotic properties, transitioning to a pro-inflammatory and pro-thrombotic phenotype [70]. Injured endothelial cells activate transcription factors such as NF-κB, upregulating the expression of a series of inflammation-related genes [73]. The products of these genes, including adhesion molecules (e.g., VCAM-1), chemokines (e.g., MCP-1 and fractalkine), and procoagulant factors (e.g., TF, VWF, and PAI-1), recruit monocytes, T lymphocytes, and other inflammatory cells, promoting their accumulation within the vessel wall (Figure 2) [74,75]. These inflammatory cells release additional inflammatory mediators, perpetuating a vicious cycle and exacerbating the inflammatory response within the vessel wall.

NF-κB is a pivotal transcription factor that plays a central role in the development of atherosclerosis (Figure 2) [76]. In endothelial cells, various pro-inflammatory stimuli, including IL-1, TNF, endotoxin, oxidized low-density lipoprotein, and advanced glycation end products, synergistically activate the NF-κB signaling pathway [77,78]. Additionally, alterations in hemodynamic factors, such as turbulence and low shear stress, influence NF-κB activity, further contributing to atherosclerosis development [79,80]. Endothelial cell activation can be classified into type I and type II activation [81]. Type I activation is an acute response that is typically triggered by infection or injury, leading to increased vascular permeability and leukocyte infiltration. In contrast, type II activation is a chronic response characterized by the sustained expression of inflammatory molecules in endothelial cells, and it results in a chronic inflammatory environment that promotes the formation and growth of atherosclerotic plaques. The cellular network within the vessel wall—including endothelial cells, smooth muscle cells, monocytes/macrophages, and keratinocytes—releases cytokines, growth factors, and ROS via paracrine signaling, further amplifying the inflammatory response [82]. This intricate cellular interplay drives the progression of atherosclerosis. Endothelial cell injury is the initiating event in atherosclerosis, with NF-κB activation serving as a critical step. Inflammation is a persistent core pathological feature throughout the disease process. A comprehensive understanding of these mechanisms is essential for developing more effective treatments to delay or reverse the progression of CVDs.

#### 2.3.2. Oxidative Stress in the Formation of Atherosclerotic Lesions

Atherosclerosis is a chronic, progressive disease driven by endothelial injury, inflammation, lipid deposition, and plaque formation within the arterial intima. It is a major contributor to CVD [83], but it represents only one aspect of the multifactorial etiology underlying these conditions. After activation by ROS, MMPs can induce degradation of elastic fibers in the vascular wall and vascular dilation, ultimately leading to the formation or dissection of aortic aneurysms [84]. These mechanisms not only impair vascular function but also exacerbate atherosclerosis and its associated complications. The pathological process of atherosclerosis follows a series of key events: (1) lipoprotein oxidation induces endothelial injury, which triggers inflammation and the recruitment of monocytes; (2) these monocytes differentiate into macrophages, which then engulf oxidized low-density lipoprotein (ox-LDL), forming foam cells; (3) the accumulation of foam cells leads to the formation of fatty streaks, which may progress to the development of a fibrous cap; and (4) plaque rupture exposes the thrombogenic core, resulting in thrombosis and potential vessel occlusion [85,86,87].

Macrophages can be polarized into M1 and M2 phenotypes. M1 macrophages primarily participate in Th1 cell-mediated inflammatory responses, secreting abundant pro-inflammatory cytokines, chemokines, nitric oxide, and ROS to eliminate pathogens [88]. In contrast, M2 macrophages exhibit anti-inflammatory properties and are involved in tissue repair, angiogenesis, and other processes. They secrete anti-inflammatory cytokines such as IL-10 and TGF-β to suppress inflammation [89]. Studies have shown that foam cells respond differently to M1- and M2-polarizing stimuli. When exposed to M1-polarizing factors (e.g., LPS and IFN-γ), foam cells exhibit a weaker pro-inflammatory response compared to normal macrophages [90]. Conversely, when exposed to M2-polarizing factors (e.g., IL-4), foam cells show a similar anti-inflammatory response to normal macrophages [90].

Foam cells are pivotal components of atherosclerotic plaques that are primarily derived from macrophages but sometimes originate from vascular endothelial and smooth muscle cells [91]. These cells undergo phenotypic switching under specific conditions, contributing to plaque formation. Foam cell formation is critically dependent on lipid accumulation, particularly the uptake of ox-LDL [92]. Nox5 promotes plaque progression by inducing LDL oxidation and foam cell formation. Simultaneously, ROS generated by Nox5 activates matrix metalloproteinases (MMPs), impairing plaque stability and exacerbating the lesion [93]. Macrophages internalize ox-LDL through scavenger receptors such as SR-A1, CD36, and LOX-1 and esterify cholesterol using enzymes like ACAT1, leading to intracellular lipid accumulation and foam cell formation [94]. During atherosclerosis, macrophage cholesterol homeostasis is disrupted: lipid uptake is enhanced by the upregulation of receptors like LOX-1, while cholesterol efflux is impaired due to the downregulation of transporters such as ABCA1 and ABCG1 [95]. Pro-inflammatory cytokines, ox-LDL, lysophosphatidylcholine, and other factors synergistically promote macrophage-to-foam cell transformation, exacerbating plaque development [96]. Foam cell formation is a hallmark of atherosclerosis, driven by the dysregulation of lipid metabolism, inflammation, and phenotypic switching. A comprehensive understanding of foam cell formation mechanisms is essential for developing novel therapeutic strategies against atherosclerosis.

NLRP3 plays a pivotal role in the development of atherosclerosis [97]. As a member of the NLR family, NLRP3 (nucleotide-binding oligomerization domain, leucine-rich repeat, and pyrin domain containing 3) is a key component of the inflammasome [98]. The activation of NLRP3 is a multi-step process involving multiple signaling pathways. Pathogen-associated molecular patterns (PAMPs) and damage-associated molecular patterns (DAMPs) can recognize and bind to NLRP3, inducing conformational changes [99]. Simultaneously, these stimuli can activate other signaling pathways, such as the NF-κB pathway, to promote the expression of NLRP3 and pro-inflammatory cytokines like IL-1β [100]. Furthermore, alterations in intracellular environments, such as decreased potassium ion concentrations, ROS generation, and lysosomal dysfunction, can directly or indirectly activate NLRP3 [101]. Upon activation, NLRP3 recruits the apoptosis-associated speck-like protein containing CARD (ASC) and caspase-1, forming a complex that cleaves pro-IL-1β and pro-IL-18 into their active forms, triggering a robust inflammatory response [102]. The release of pro-inflammatory cytokines IL-1β and IL-18 induced by NLRP3 activation promotes the formation and development of atherosclerotic plaques [103]. Additionally, NLRP3 can polarize macrophages toward the M1 phenotype, which exhibit a pro-inflammatory phenotype, exacerbating atherosclerosis [104]. It can also promote the dedifferentiation and migration of vascular smooth muscle cells, leading to plaque instability [105]. Furthermore, NLRP3 can enhance cholesterol uptake and esterification, promoting the formation of foam cells [106].

#### 2.3.3. Oxidative Stress and Reperfusion Injury in Acute Ischemic Events

Oxidative stress plays a pivotal role in ischemia-reperfusion injury during acute cardiovascular events such as myocardial infarction and stroke. Acute myocardial ischemia leads to a severe oxygen shortage in the myocardium. Under hypoxic conditions, myocardial cell metabolism shifts from aerobic respiration to anaerobic glycolysis, leading to reduced ATP production, lactate accumulation, and a decrease in intracellular PH [107].The acidic microenvironment further exacerbates cell injury by inhibiting mitochondrial permeability transition pore (mPTP) opening and disrupting ion homeostasis, ultimately causing cardiomyocyte death and severe cardiac dysfunction [108]. Additionally, even under low-flow ischemia or in the presence of residual oxygen, myocardial tissue can produce large amounts of ROS, further aggravating oxidative stress and accelerating myocardial injury [109].

Myocardial ischemia-reperfusion injury (MIRI) plays a pivotal role in the pathogenesis of heart disease [110]. Studies have demonstrated that ROS not only trigger a cascade of cellular events but also directly activate the NLRP3 inflammasome, playing pivotal roles in the pathogenesis of myocardial ischemia-reperfusion injury (MIRI) [111,112,113]. The activated NLRP3 inflammasome subsequently leads to the release of inflammatory cytokines, inducing a robust inflammatory response [114,115]. Moreover, the NLRP3 inflammasome plays a multifaceted role in MIRI, contributing to both cell death and endothelial dysfunction, resulting in vasoconstriction, and exacerbating cardiac injury [114].

Multiple factors contribute to the generation of ROS during myocardial ischemia-reperfusion injury, including the mitochondrial respiratory chain, xanthine oxidase, NADPH oxidase, nitric oxide synthase, and endoplasmic reticulum stress [116]. ROS generated during reperfusion oxidatively damage mitochondrial membrane proteins, phospholipids, and both mitochondrial (mtDNA) and nuclear (nDNA) genomes, thereby disrupting mitochondrial structure and function [117]. As the cell’s powerhouse, mitochondria provide energy for cardiomyocytes (HCMs), and impaired mitochondrial function directly impacts energy supply, weakening cardiac contractility and relaxation and ultimately contributing to CVDs. Mutations in mtDNA and nDNA further exacerbate mitochondrial dysfunction, resulting in excessive ROS production, decreased oxidative phosphorylation (OXPHOS) capacity, and a cascade of pathophysiological changes [118]. Mitophagy, a selective form of autophagy, serves as a quality control mechanism by degrading damaged mitochondria to maintain cellular homeostasis [119]. Impaired mitophagy leads to the accumulation of dysfunctional mitochondria, and BMAL1-mediated mitochondrial dysfunction is closely linked to dilated cardiomyopathy [120]. In myocardial ischemia-reperfusion injury, casein kinase 2α exacerbates cardiomyocyte injury by inhibiting mitophagy [121]. The induction of mitophagy can effectively alleviate mitochondrial dysfunction and lipid accumulation induced by a high-fat diet, preventing diabetic cardiomyopathy [122]. Decreased mitochondrial OXPHOS and increased ROS production are pivotal in the pathogenesis of CVDs. ROS damage mitochondrial DNA, induce apoptosis, and promote inflammation. In chronic heart failure and aneurysms, decreased OXPHOS function has been linked to reduced succinyl-CoA levels, decreased mitochondrial fusion, and loss of mitochondrial membrane potential [123]. Additionally, transcription factors like *PGC-1β* and microRNAs such as *miR-27b-3p* regulate OXPHOS, and their dysregulation is closely associated with CVDs [124].

#### 2.3.4. Oxidative Stress Mechanisms in Cardiac Remodeling and Chronic Heart Failure

Heart failure (HF) is characterized by cardiac remodeling, which involves alterations in cardiomyocyte structure and function and remodeling of the extracellular matrix [125]. These changes result in abnormal cardiac geometry, ventricular dilation, and impaired contractile function, ultimately compromising cardiac output. Numerous studies have demonstrated elevated levels of ROS in HF patients, highlighting the significant role of oxidative stress in the pathogenesis and progression of HF [126,127]. Both rapid pacing-induced canine HF models and murine myocardial infarction (MI) models recapitulate the pathophysiological features of human HF, exhibiting remarkable similarities in cardiac structure, function, and hemodynamics [128]. Notably, studies have shown that knockout of the *p47phox* gene in mice attenuates post-MI cardiac remodeling and dysfunction, suggesting a crucial role for NAD(P)H oxidase in post-MI heart disease. Furthermore, other studies have provided direct evidence that angiotensin II exacerbates cardiac injury by activating NAD(P)H oxidase in vascular endothelial cells, leading to mitochondrial dysfunction [128].

ROS activate multiple signal transduction pathways, including MAPK/ERK, JNK, and p38, leading to the phosphorylation of downstream transcription factors that induce the synthesis of structural proteins in cardiomyocytes and contributing to cardiac hypertrophy [129]. Concurrently, ROS can trigger the caspase cascade, resulting in cardiomyocyte apoptosis through mitochondrial damage and endoplasmic reticulum stress. Additionally, ROS activate matrix metalloproteinases (MMPs) such as MMP-2 and MMP-9, which degrade the extracellular matrix, disrupt the myocardial microenvironment, and drive ventricular remodeling [130]. Moreover, ROS activate transcription factors like nuclear factor-κB (NF-κB), inducing the expression of inflammatory factors such as tumor necrosis factor-α (TNF-α) and interleukin-6 (IL-6), triggering inflammatory responses, and exacerbating myocardial injury [131]. Disruption of calcium homeostasis and endothelial cell injury are also detrimental effects of ROS on the heart. These mechanisms create a vicious cycle, leading to the progressive deterioration of cardiac structure and function, ultimately promoting pathological cardiac remodeling and contributing to terminal conditions like heart failure.

#### 2.3.5. Protective Roles of ROS in CVDs

Although excessive ROS generation can induce oxidative stress and cellular damage during ischemia-reperfusion injury (IRI), the protective role of moderate levels of ROS in IRI has been extensively studied, primarily in terms of inducing cytoprotective mechanisms and modulating signaling pathways [132].

ROS can induce myocardial preconditioning and postconditioning. Low levels of ROS generated during ischemia activate protective signaling pathways (such as PI3K/Akt, AMPK) and redox-sensitive transcription factors (such as Nrf2), enhancing antioxidant capacity and inhibiting apoptotic gene expression [133]. This “adaptive” effect reduces myocardial cell injury during subsequent ischemia-reperfusion. Postconditioning exerts its cardioprotective effects by activating multiple signaling cascades, such as the mK (ATP) and PKC pathways, and by regulating ROS levels [134]. These findings suggest that these signaling pathways are essential components of postconditioning-induced cardiovascular protection [135]. Early ROS release upon reperfusion induces endogenous protective mechanisms, such as activation of mitochondrial ATP-sensitive potassium (KATP) channels, heat shock protein (HSP) expression, and upregulation of anti-apoptotic factors, thereby attenuating IRI-induced apoptosis [136,137].

ROS generation can modulate redox-sensitive signaling pathways. Moderate levels of ROS can oxidize Keap1 protein, leading to the release and nuclear translocation of Nrf2, promoting the expression of antioxidant enzymes (such as SOD, CAT, and HO-1), enhancing cellular antioxidant capacity, and alleviating inflammation and oxidative damage in IRI [138]. ROS-mediated PI3K/Akt activation can regulate downstream eNOS generation of NO, improving microcirculation, inhibiting cardiomyocyte apoptosis, and exerting cardioprotective effects [139]. Moderate ROS can enhance mitochondrial autophagy through the AMPK pathway, maintaining mitochondrial function stability and reducing mitochondrial damage caused by IRI.

ROS generation can regulate mitochondrial function. Moderate increases in ROS levels can promote mitochondrial fusion (by upregulating fusion proteins such as OPA1), improve energy supply, and reduce oxidative stress by removing damaged mitochondria [140]. The ROS-induced opening of KATP channels can alleviate mitochondrial calcium overload, reduce membrane potential collapse, and protect mitochondrial function [141,142].

The effects of ROS exhibit a clear dose-dependent relationship. Low concentrations of ROS can serve as cellular signaling molecules, participating in various physiological processes and exerting protective effects [143]. For example, low concentrations of ROS can activate the Nrf2 pathway, inducing the expression of antioxidant enzymes and protecting cells from oxidative damage. High concentrations of ROS, however, can induce oxidative stress, leading to lipid peroxidation, protein denaturation, DNA damage, and ultimately cellular damage, inflammation, and cardiovascular diseases [144].

The protective effects of ROS in ischemia-reperfusion injury are achieved through inducing myocardial preconditioning, activating protective signaling pathways (such as Nrf2 and PI3K/Akt), regulating mitochondrial function, and maintaining redox balance. Future research should focus on precisely regulating ROS levels to maximize its protective effects, providing new strategies for the treatment of IRI.

## 3. The Protective Effects of Antioxidants and the Underlying Redox Signaling Mechanisms in the Heart

### 3.1. Redox Signaling Pathways

Redox signaling pathways are crucial for cardiovascular protection, as they regulate ROS and reactive nitrogen species (RNS) levels, modulating cellular processes to mitigate oxidative stress-induced cardiomyocyte injury. This section explores these essential pathways.

#### 3.1.1. Nrf2/Keap1/ARE Signaling Pathway

The Nrf2/Keap1/ARE signaling pathway is a finely tuned regulatory system that orchestrates cellular responses to oxidative stress and chemical attacks [145]. *Nrf2*, a central transcription factor, binds to antioxidant response elements (*AREs*) to regulate the expression of genes involved in antioxidant defense and detoxification [146]. *Keap1*, a negative regulator of *Nrf2*, sequesters *Nrf2* in the cytoplasm, inhibiting its transcriptional activity. ROS generated by Nox5 activation can upregulate the expression of antioxidant genes via the Nrf2/Keap1 pathway while simultaneously activating inflammatory responses through the NF-κB pathway, further exacerbating oxidative stress [147]. Under oxidative stress or ionizing radiation, *Keap1* undergoes modifications that disrupt its interaction with *Nrf2*, allowing *Nrf2* to translocate to the nucleus and activate target gene expression [148].

In ischemia-reperfusion injury (IRI) conditions, Nrf2 activation induces the expression of antioxidant enzymes, such as superoxide dismutase (SOD), glutathione peroxidase (GPx), and glutathione reductase (GR), which scavenge excess ROS and mitigate oxidative damage. Additionally, Nrf2 interacts with the NF-κB signaling pathway to suppress inflammatory responses and alleviate tissue injury. Moreover, Nrf2 activates heme oxygenase-1 (HO-1) expression, promoting the degradation of heme into bilirubin, an antioxidant that provides further cellular protection [113].

In doxorubicin (DOX)-induced oxidative stress, DOX treatment upregulates Keap1 expression and inhibits Nrf2 activity through multiple mechanisms, including recruitment of TRIM21, which interferes with the separation of Nrf2 from Keap1, thereby weakening the cell’s antioxidant defense [149]. Sirtuin 1 (Sirt1) activates Nrf2 through deacetylation, enhancing its transcriptional activity. Conversely, miR-140-5p exacerbates oxidative stress by targeting Sirt2 and inhibiting Nrf2 activation. Other proteins, such as p62/Sqstm1, PKC, PI3K/Akt, and p21, regulate Nrf2 activity through various mechanisms [150,151]. Several natural compounds, including dioscin, α-linolenic acid, and sulforaphane, have been shown to enhance myocardial antioxidant capacity and alleviate DOX-induced myocardial injury by activating the Nrf2/Keap1/ARE pathway [152,153,154]. Nrf2 holds promise as a therapeutic target for DOX-induced myocardial injury, and the development of drugs that activate the Nrf2 pathway is a promising therapeutic strategy.

Type 2 diabetes mellitus (T2DM) is a major risk factor for CVD patients globally. Studies have demonstrated that individuals with diabetes are at a significantly higher risk of developing CVDs compared to those without diabetes, with this risk being positively correlated with blood glucose levels. Diabetic patients frequently exhibit cardiac damage, and even when blood glucose levels are well-controlled, the risk of cardiovascular events remains elevated relative to the general population [155]. Hinokinin, a natural compound with various pharmacological properties, has been shown to reduce cardiac oxidative stress, inflammation, and apoptosis induced by T2DM. This effect occurs through modulation of the Nrf2/Keap1/ARE pathway and inhibition of the TLR4/MyD88/NF-κB and MAPK signaling pathways, ultimately protecting the myocardium [156]. Moreover, *Nrf2* activators have been found to mitigate diabetes-induced endothelial dysfunction (ED) [157]. Our in vitro study using high-glucose-induced HK-2 cells demonstrated that chitosan (COS) exerts antioxidant effects by activating the Nrf2/Keap1/ARE pathway, providing experimental evidence for its potential therapeutic value in diabetic complications [158].

#### 3.1.2. PI3K/Akt Pathway

The PI3K/Akt pathway stands as a cornerstone of cardiomyocyte survival, orchestrating cardiovascular protection through the precise modulation of cellular growth, proliferation, and apoptosis [159]. Through the activation of the mTOR pathway, PI3K/Akt enhances protein synthesis and cellular metabolism, promoting angiogenesis and ensuring adequate myocardial oxygenation and nutrient supply. Additionally, this pathway exerts anti-inflammatory effects, reducing myocardial injury [160].

Activation of the PI3K/Akt pathway has been shown to significantly mitigate myocardial injury induced by myocardial infarction (Table 1). Both human and animal studies have demonstrated a significant upregulation of PI3K and Akt activity post-myocardial infarction. Inhibition of the PI3K/Akt pathway exacerbates cardiovascular risk and increases mortality in mice, whereas activation of this pathway by inhibiting PTEN improves cardiac function. These findings collectively underscore the therapeutic potential of targeting the PI3K/Akt pathway in myocardial infarction [161,162].

While the activation of the PI3K/Akt pathway provides protection in the early stages of acute heart failure, prolonged excessive activation can lead to myocardial remodeling and functional deterioration [163]. This pathway is involved in myocardial remodeling processes such as cardiomyocyte hypertrophy, fibrosis, and apoptosis. Chronic inflammation, a hallmark of heart failure, can be mitigated by activated protein C (APC), which inhibits the PI3K/Akt pathway [164]. Regarding angiogenesis, APC plays a dual role: while the PI3K/Akt pathway primarily promotes angiogenesis, the interaction between the two may influence angiogenesis outcomes in heart failure. Furthermore, APC can induce apoptosis in certain cells, while the PI3K/Akt pathway inhibits apoptosis. The balance between these two pathways determines cell fate in heart failure [165].

#### 3.1.3. AMPK Pathway

AMP-activated protein kinase (AMPK), a key cellular energy sensor, plays a crucial role in maintaining cellular energy homeostasis (Table 1). In the cardiovascular system, AMPK activation exerts cardioprotective effects by promoting mitochondrial bioenergetics, enhancing fatty acid oxidation, activating antioxidant defense systems, and regulating glucose metabolism. In CVDs such as myocardial IRI and HF, AMPK activation attenuates cardiomyocyte apoptosis, inhibits inflammation, and improves cardiac function. AMPK is activated under oxidative stress conditions and exerts its cardioprotective effects by regulating downstream effectors such as peroxisome proliferator-activated receptor gamma coactivator-1α (PGC-1α) and acetyl-CoA carboxylase (ACC).

In atherosclerosis, risk factors such as hyperglycemia and hyperlipidemia induce excessive mitochondrial ROS production, leading to endothelial cell damage [166]. AMPK exerts potent vascular protective effects by inhibiting mitochondrial ROS generation, improving mitochondrial function, attenuating inflammation, and promoting endothelial cell survival [167]. Therefore, activation of AMPK holds promise as a novel therapeutic strategy for atherosclerosis.

AMPK also plays a protective role in coronary heart disease, and its activation is considered a promising therapeutic approach [168]. Researchers are currently developing various AMPK activators, such as metformin and 5-aminoimidazole-4-carboxamide ribonucleotide (AICAR), for the treatment of coronary heart disease [169]. When cellular energy levels decrease, the AMP/ATP ratio increases, activating AMPK. AMPK activation occurs primarily through several mechanisms: LKB1 phosphorylation, where LKB1, an upstream kinase of AMPK, activates AMPK by phosphorylating Thr172 on the α subunit; calcium/calmodulin-dependent protein kinase kinase β (CaMKKβ) phosphorylation, where CaMKKβ is activated upon calcium ion elevation and phosphorylates AMPK; and phosphorylation by other kinases, including TAK1 and TAK11, which can also phosphorylate AMPK [170]. AMPK is a potential therapeutic target for coronary heart disease that warrants further investigation.

#### 3.1.4. SIRT1 Pathway

SIRT1, an NAD^+^-dependent deacetylase initially identified in yeast, primarily localizes to the nucleus where it regulates gene expression and influences a variety of biological processes, including cell growth, apoptosis, and metabolism [171]. In the vascular system, SIRT1 expression and activity are closely associated with vascular homeostasis [172]. It maintains vascular homeostasis by regulating multiple downstream target proteins, such as eNOS, p53, and FOXO [173,174]. SIRT1 protects vascular endothelial cells, inhibits the proliferation of vascular smooth muscle cells, and delays vascular senescence (Table 1).

In atherosclerosis, SIRT1 exhibits therapeutic potential. To investigate its impact on atherosclerosis progression, SIRT1 was overexpressed specifically in endothelial cells of *ApoE^−/−^* mice [175]. The results demonstrated that SIRT1 overexpression ameliorates endothelial dysfunction by activating the eNOS signaling pathway and suppressing endothelial inflammation, thereby attenuating atherosclerosis development. Oral administration of low-dose red wine significantly upregulates the expression of eNOS and SIRT1 in the aortas of hypercholesterolemic mice, suggesting that resveratrol may prevent atherosclerosis through the activation of the SIRT1 pathway [176].

Studies have shown that SIRT1 expression decreases with age in human vascular smooth muscle cells and is lower in atherosclerotic lesions, suggesting that SIRT1 plays an important role in maintaining vascular smooth muscle cell function [177]. Additionally, miR-34a is highly expressed in the aortas of aged mice, where it promotes senescence and inflammation by downregulating SIRT1. This suggests that SIRT1 may play a crucial role in regulating the inflammatory responses of monocytes/macrophages [178]. Donato et al. found that SIRT1 expression is decreased in the endothelial cells of the elderly and is associated with reduced NO production by eNOS and endothelial dysfunction [179]. This indicates that SIRT1 plays an important role in maintaining endothelial function. Meanwhile, Bai et al. demonstrated that SIRT1 inhibits endothelial cell senescence by deacetylating LKB1 and promoting its degradation [180]. They also found that loss of SIRT1 expression or function leads to increased nuclear accumulation of acetylated LKB1, resulting in alterations in vascular wall structure, arterial remodeling, and vascular stiffness [181]. These findings suggest that SIRT1 plays a crucial role in maintaining vascular health. Decreased SIRT1 expression is closely associated with vascular aging and atherosclerosis. SIRT1 exerts its protective effects through multiple mechanisms, including regulating eNOS activity, inhibiting endothelial cell senescence, and exerting anti-inflammatory effects [172].

#### 3.1.5. MAPK Pathway

The MAPK pathway is a crucial intracellular signaling cascade that transmits extracellular stimuli to the nucleus, initiating a variety of cellular responses (Table 1). Typically composed of a three-kinase module—MAPKKK, MAPKK, and MAPK—the MAPK pathway regulates numerous cellular processes, including proliferation, differentiation, and apoptosis. Distinct MAPK subfamilies (ERK, JNK, and p38) target specific downstream effectors [182].

In the commonly used animal model for heart failure, transverse aortic constriction (TAC), activation of MAPK signaling pathways in cardiac tissue has been extensively studied [183]. Results consistently show that the JNK pathway is activated first, followed by the ERK and p38 MAPK pathways, suggesting a temporal hierarchy in the activation of these signaling cascades [184].

The ERK pathway plays a pivotal role in promoting cardiac hypertrophy, as evidenced by the effective suppression of hypertrophy with MEK or Raf inhibitors [185]. Grb2, an upstream regulator of the ERK pathway, is essential for ERK activation. Mice with haploinsufficiency of *Grb2* exhibit resistance to pressure overload-induced cardiac hypertrophy [186]. Cardiac-specific overexpression of a dominant-negative Raf-1 or knockout of the Raf gene can also confer resistance to cardiac hypertrophy [187]. While ERK1/2 is crucial for cardiac hypertrophy, its complete deletion or reduced activity does not fully suppress hypertrophy, suggesting the involvement of other signaling pathways [188]. In contrast, the JNK and p38 pathways inhibit cardiac hypertrophy. Loss or reduced activity of JNK1/2 or p38α leads to spontaneous cardiac hypertrophy, indicating their inhibitory roles [189]. Both JNK and p38 inhibit cardiac hypertrophy by phosphorylating and inactivating the NFAT transcription factor. These findings highlight the complex interplay between different MAPK pathways in regulating cardiac hypertrophy.

MAPK signaling pathways also play a pivotal role in cardiac remodeling following myocardial infarction [190]. Among them, p38 MAPK, the most extensively studied member, is rapidly activated after myocardial infarction and promotes pathological cardiac remodeling through multiple mechanisms. For instance, p38 MAPK induces cardiomyocyte apoptosis by downregulating anti-apoptotic proteins such as Bcl-xL and Bcl-2, thereby expanding the infarct size. Additionally, it promotes collagen synthesis, leading to cardiac fibrosis, and inhibits cardiomyocyte proliferation, hindering myocardial repair and regeneration. JNK acts synergistically with p38 MAPK to promote cardiomyocyte apoptosis and fibrosis [191]. The ASK1-JNK signaling pathway plays a critical role in this process [192]. In contrast, the role of the ERK pathway in post-myocardial infarction remodeling is more complex, potentially involving both cell survival and proliferation, although the underlying mechanisms require further investigation.

#### 3.1.6. CVDs Associated with Oxidative Stress and Their Underlying Signaling Pathways

Oxidative stress plays a pivotal role in promoting the progression of various CVDs by activating a series of signaling pathways. This table comprehensively summarizes the critical roles of oxidative stress in different CVDs, detailing the major signaling pathways involved and the specific impacts of oxidative stress on pathological processes. For example, in atherosclerosis, oxidative stress activates the NF-κB signaling pathway, leading to chronic inflammation and plaque formation. In dilated cardiomyopathy, ROS accumulation induces apoptosis and myocardial remodeling, ultimately leading to heart failure.

**Table 1:** Key signaling pathways and their pathological effects in oxidative stress-related CVDs.

CVDs	Core Signaling Pathways and Their Mechanisms		Role of ROS in CVDs	Reference
AS	NF-κB Nrf2 MAPK PI3K/Akt SIRT1	NF-κB drives inflammation, Nrf2 promotes antioxidant defense, SIRT1 regulates metabolism, and MAPK/Akt promotes proliferation.	Increases lipid oxidation promotes plaque formation and progression.	[193,194,195]
HTN	RAAS ROS ET-1 NOX AMPK	RAAS increases vascular tension, NOX generates ROS, AMPK regulates energy metabolism and improves vascular function, and ET-1 promotes vasoconstriction.	Leads to vascular stiffening, promoting increased vascular resistance and endothelial dysfunction.	[196,197]
CAD	NF-κB p38 MAPK PI3K/Akt SIRT3	p38 MAPK regulates inflammation, SIRT3 promotes antioxidant defense, and NF-κB induces inflammation.	Induces endothelial cell apoptosis, promoting coronary plaque instability.	[198,199]
AMI	JAK/STAT NF-κB p38 MAPK HIF-1α	HIF-1α activates protective genes, JAK/STAT regulates inflammation, and p38 MAPK promotes cardiac repair.	Results in cardiomyocyte apoptosis and necrosis, leading to aggravated myocardial injury.	[200,201]
DCM	TGF-β PI3K/Akt JNK ERK	TGF-β promotes fibrosis, JNK/ERK induces injury, and PI3K/Akt prevents apoptosis.	Exacerbates cardiac fibrosis, leading to ventricular remodeling and functional failure.	[202,203]
HCM	IGF-1 mTOR ERK GATA4	GATA4 induces hypertrophic genes and IGF-1/mTOR promotes growth.	Exacerbates cardiac hypertrophy and oxidative stress, leading to cardiac dysfunction.	[204]
PH	ET-1 TGF-β NFAT PI3K/Akt mTOR	NFAT and TGF-β induce vascular smooth muscle cell proliferation and remodeling and mTOR regulates cell proliferation.	Induces vascular smooth muscle cell proliferation and increases pulmonary arterial pressure.	[205]
MIRI	ROS JAK/STAT ERK1/2 NOX	NOX induces oxidative damage and ERK1/2 regulates cell growth and repair.	Induces cardiomyocyte apoptosis and necrosis, leading to reperfusion injury.	[206,207]
CVI	NOX NF-κB VEGF MMP	MMPs degrade the extracellular matrix, facilitating vascular remodeling, and VEGF promotes angiogenesis.	Increases inflammation and vascular permeability, leading to chronic vascular wall injury.	[208,209]
VTE	P-selectin TGF-β NF-κB COX-2	COX-2 promotes inflammation and P-selectin mediates leukocyte and platelet adhesion.	Promotes thrombus formation and exacerbates vascular occlusion.	[210,211]
RHD	JAK/STAT NF-κB IL-17	IL-17 drives chronic inflammation and induces immune responses.	Induces chronic inflammation and fibrosis, impairing cardiac valve function.	[212]
CAV	NF-κB mTOR JAK/STAT PD-1/PD-L1	PD-1/PD-L1 regulates immune suppression and mTOR regulates cell proliferation.	Promotes chronic rejection and vascular remodeling, leading to allograft heart injury.	[213,214]
Afib	CaMKII NF-κB TGF-β	TGF-β induces fibrosis and atrial remodeling and CaMKII modulates myocardial electrical remodeling.	Leads to atrial remodeling, increasing the incidence of atrial fibrillation.	[215]
HF	β-AR PI3K/Akt NF-κB Notch	Notch signaling modulates cell proliferation and apoptosis and β-AR potentiates cardiac contractility.	Increases apoptosis and fibrosis, leading to impaired cardiac function.	[216,217]
Heart inflammation	TLR NF-κB MAPK IL-1β	IL-1β mediates the inflammatory response and TLRs mediate the recognition of pathogens and trigger immune responses.	Induces oxidative stress and immune responses, thereby exacerbating cardiac injury.	[218,219]
Rheumatic carditis	NF-κB TGF-β JAK/STAT IL-6	IL-6 drives chronic inflammation and JAK/STAT signaling modulates immune cell activation.	Induces chronic myocardial inflammation, thereby exacerbating cardiac injury.	[220,221]
MVP	TGF-β MMP NF-κB SMAD	Smad signaling contributes to valvular fibrosis by promoting extracellular matrix protein synthesis.	Increases valvular fibrosis, thereby impairing valve function.	[222,223]
HOCM	CaMKII ROS PI3K/Akt ERK	ERK mediates cardiac hypertrophy and PI3K/Akt regulates cell survival and proliferation.	Exacerbates myocardial hypertrophy and dysfunction.	[224,225]
CHD	Wnt/β-catenin Notch NF-κB SHH	Wnt and SHH signaling regulate cardiac development and Notch regulates cell differentiation.	Causes developmental abnormalities, thereby exacerbating congenital heart disease.	[226,227]
Coronary artery spasm angina	PKC eNOS ET-1 CaMKII	CaMKII affects myocardial contractility and eNOS regulates nitric oxide production.	Causes coronary artery constriction, leading to ischemic events.	[228]
PSVT	CaMKII PKA RyR SERCA	SERCA regulates calcium ion reuptake and RyR regulates calcium ion release.	Causes arrhythmia, thereby increasing myocardial workload.	[229]
CHF	β-AR ROS NF-κB SIRT3	SIRT3 protects mitochondrial function and reduces oxidative stress.	Promotes myocardial fibrosis and apoptosis, leading to aggravated heart failure.	[230]
Left ventricular insufficiency	TGF-β NF-κB PI3K/Akt IL-1β	IL-1β induces cardiac fibrosis and TGF-β triggers structural remodeling.	Causes myocardial fibrosis.	[231]

**AS:** Atherosclerosis; **HTN:** Hypertensive; **CAD:** Coronary heart disease; **AMI**: Acute myocardial infarction; **DCM:** Dilated cardiomyopathy; **HCM:** Hypertrophic cardiomyopathy; **PH:** Pulmonary arterial hypertension; **MIRI:** Myocardial ischemia-reperfusion injury; **CVI:** Chronic venous insufficiency; **VTE:** Venous thromboembolism; **RHD:** Rheumatic heart disease; **CAV:** Cardiac allograft vasculopathy; **Afib:** Atrial fibrillation; **HF:** Heart failure; **MVP:** Mitral valve prolapse; **HOCM:** Hypertrophic obstructive cardiomyopathy; **CHD:** Congenital heart disease; **PSVT:** Paroxysmal supraventricular tachycardia; and **CHF:** Congestive heart failure.

### 3.2. Antioxidants

Antioxidants have been extensively studied for their potential to mitigate oxidative damage in CVDs (Table 2). These compounds exert their effects through various mechanisms, including free radical scavenging, inhibition of lipid peroxidation, suppression of apoptosis, and modulation of redox signaling. Additionally, diverse animal models have been employed to investigate the efficacy and safety of different antioxidants in simulating human cardiovascular pathologies (Table 2).

The following table summarizes the major antioxidants currently under investigation, their applications in various CVDs, and their underlying mechanisms of action. Additionally, commonly used animal models are listed, providing a comprehensive overview of the potentials of different antioxidants in cardiovascular protection.

**Table 2:** Applications and Mechanisms of Antioxidants in CVDs: A Focus on Animal Models.

Antioxidant	Main Mechanism of Action	CVDs Applied	Animal Model	Reference
Vitamin C	Antioxidant scavenging of free radicals, reduction of lipid peroxidation, protection of endothelial function, and inhibition of inflammatory response.	AS CHD HTN	*LDLr^−/−^* mice	[232]
Vitamin E	Inhibits lipid peroxidation, reduces oxidative damage, and inhibits inflammation and cell proliferation.	AS MIRI	CETP transgenic rats	[233]
Glutathione	Increases antioxidant reserves, regulates redox balance, and attenuates mitochondrial damage.	HTN CHD Myocardial fibrosis	Aldosterone-induced hypertension in C57BL/6 mice	[234]
lipoic acid	Inhibits oxidative stress and inflammatory responses and improves mitochondrial function.	DbCM HTN HF	RAS-activated mice	[235]
CoQ10	Enhances mitochondrial respiratory chain function, reduces ROS production, and improves myocardial tolerance.	HF MIRI AS	*ApoE^−/−^* mice	[236]
Resveratrol	Activates SIRT1, reduces oxidative stress, and inhibits inflammation.	MIRI AS	*ApoE^−/−^* mice	[237]
N-Acetylcysteine	Provides GSH precursors, reduces ROS, and enhances antioxidant capacity.	CHD MIRI	MIRI in C57BL/6 Mice	[238]
Astaxanthin	A potent antioxidant that reduces lipid peroxidation and inhibits inflammatory responses.	MIRI HF AS	SOD2-deficient mice	[239]
Quercetin	Inhibits oxidative stress and lipid peroxidation and enhances anti-inflammatory capacity.	HTN AS CHD	AT1 transgenic mice	[240]
Tea polyphenols	Inhibits endothelial inflammation and lipid peroxidation and protects the myocardium and blood vessels.	Diabetic AS CMP	Leprdb/db mice	[241]
Sodium thiosulfate	Scavenges ROS, reduces mitochondrial oxidative stress, and inhibits calcium overload and apoptosis.	MIRI HF	*SIRT3* gene-deficient mice	[242]
Statin drugs	Reduces cholesterol, inhibits ROS production, and attenuates vascular endothelial damage.	AS CHD HTN	*LDLr^−/−^* and *ApoE^−/−^* mice	[243]
Vitamin D	Regulates calcium metabolism, reduces vascular smooth muscle cell proliferation, and protects the endothelium.	HTN AS Myocardial Fibrosis	HTN-induced *ApoE^−/−^* mice	[244]
Gallic acid	Inhibits oxidative stress and smooth muscle cell proliferation and regulates endothelial cell activity.	AS DbCM	Cholesterol-hypercholesterolemic rat model	[245]
Hydrogen sulfide	Acts as an endogenous antioxidant molecule, regulates mitochondrial function, and inhibits apoptosis.	MIRI HF DbCM	Diabetes-induced ZDF in rats	[246]
Thioredoxin	Inhibits ROS generation, protects endothelial cells and mitochondria, and regulates calcium ion balance.	AS HTN HF	Angiotensin II-induced hypertension in C57BL/6 mice	[247]

**AS**: Aortic Stenosis; **CHD**: Coronary Heart Disease; **HTN**: Hypertension; **MIRI**: Myocardial Ischemia-Reperfusion Injury; **HF**: Heart Failure; **DbCM**: Diabetic Cardiomyopathy; and **CMP**: Cardiomyopathy.

## 4. Emerging Paradigms in Cardiovascular Protection

Advancements in molecular biology, gene editing, and stem cell technology have led to the development of novel cardiovascular protection strategies, bridging the gap between basic research and clinical application.

### 4.1. Drug Therapy

MitoTEMPO (MT) and Visomitin (SKQ1) have emerged as pioneering mitochondrial-targeted antioxidants, spearheading a new era in antioxidant therapy [248]. Functioning akin to precision surgical instruments, they directly target the cellular powerhouse, the mitochondria, to selectively eliminate deleterious ROS [248]. Derived from TEMPOL, MT, plastoquinone, and SKQ1, these compounds, despite their structural dissimilarities, share a common objective: safeguarding mitochondrial health [249]. The distinct structures of SKQ1 and MT confer upon them unique antioxidant mechanisms (Table 3). By leveraging the electron transfer capacity of plastoquinone, SKQ1 acts as an electronic “sponge”, rapidly absorbing and neutralizing harmful free radicals [250]. Conversely, MT mimics superoxide dismutase, efficiently calyzing the conversion of superoxide anions, functioning as a molecular “scavenger” [251]. The triphenylphosphonium moiety serves as their “navigation system”, precisely guiding them to the mitochondria. Research has demonstrated that both MT and SKQ1 can effectively scavenge free radicals, mitigate oxidative stress-induced cellular damage, and even improve cardiomyocyte function, offering promising therapeutic avenues for cardiovascular diseases (Table 3) [252]. In vitro experiments using H_2_O_2_- and MEN-induced oxidative damage models have revealed that both MT and SKQ1 exhibit significant cytoprotective effects, underscoring their potential as therapeutic agents in antioxidant therapy [253,254,255].

Nrf2, a pivotal transcription factor, plays an indispensable role in maintaining redox homeostasis (Table 3). Upon exposure to oxidative stress, Nrf2 is activated and rapidly translocates into the nucleus, where it binds to antioxidant response elements (AREs) to induce the expression of a battery composed of antioxidants and phase II detoxification enzymes [256]. Consequently, the cellular antioxidant capacity is enhanced, mitigating oxidative damage and protecting cells from further injury induced by oxidative stress [256]. *Nrf2* activators can alleviate oxidative stress-induced damage to the cardiovascular system and prevent cardiovascular diseases [257]. By upregulating the expression of antioxidant and detoxification enzymes, such as SOD, GPX, and GST, *Nrf2* activators enhance the cellular capacity to eliminate ROS and detoxify xenobiotics [258,259]. Moreover, *Nrf2* activators suppress inflammatory signaling pathways, including NF-κB, thereby mitigating inflammatory responses and protecting cells from oxidative damage [260].

Small molecule scavengers hold great promise as novel therapeutic agents for cardiovascular diseases (Table 3). By directly scavenging harmful ROS, reducing inflammation, and protecting vascular endothelial cells, these compounds offer new therapeutic options for patients with cardiovascular diseases (Table 3) [261,262,263]. They have shown great potential in slowing the progression of atherosclerosis, improving cardiac function, and reducing the risk of cardiovascular events [264].

Enzyme-based therapy is a therapeutic approach that utilizes the catalytic properties of enzymes to treat diseases. Given their high specificity and efficiency as biological catalysts, enzymes hold great promise for the treatment of cardiovascular diseases (Table 3). In cardiovascular diseases, enzymes can clear oxidized low-density lipoprotein (ox-LDL), inhibit inflammatory responses, and stabilize atherosclerotic plaques [265]. They can also scavenge ROS generated by cardiomyocytes, alleviate oxidative damage, and inhibit cardiomyocyte apoptosis (Table 3). Moreover, enzymes can improve myocardial energy metabolism and enhance myocardial contractility [266]. The therapeutic efficacy of enzymes largely depends on their delivery in vivo. Current challenges in enzyme delivery include the instability of enzymes in biological fluids and the difficulty of targeting enzymes toward disease sites [267]. To address these challenges, researchers are developing various enzyme delivery systems, such as liposomes and nanoparticles [268]. Enzyme-based therapy has great potential for the treatment of cardiovascular diseases, but challenges remain. With the continuous development of nanotechnology and genetic engineering, enzyme-based therapy is expected to become a significant modality for the treatment of cardiovascular diseases in the future.

ROS metabolism inhibitors hold great promise in the treatment of cardiovascular diseases (Table 3). By inhibiting the activity of ROS-generating enzymes, enhancing antioxidant enzyme activity, and modulating redox signaling pathways, these agents effectively scavenge excessive ROS and alleviate oxidative stress [269,270]. Consequently, ROS metabolism inhibitors can delay the progression of atherosclerosis, mitigate myocardial infarction injury, and improve symptoms of heart failure, providing a novel therapeutic strategy for cardiovascular disease patients [271].

Multifunctional antioxidants are versatile compounds that offer multiple modes of action to counteract oxidative stress (Table 3). In cardiovascular diseases, these agents can directly scavenge mitochondrial-derived ROS, chelate redox-active metals, and induce antioxidant enzymes, thereby providing significant cardiovascular protection [272].

**Table 3:** Pharmacological Approaches Targeting Oxidative Stress and Their Characteristics.

Category	Drug	Mechanism	Advantages and Innovation	Reference
**Mitochondria-Targeted Antioxidants**	**MitoTEMPO**	Targets mitochondria to reduce ROS generation and improve mitochondrial function.	Targeting mitochondria with high specificity, directly acting on major ROS production sites, and reducing side effects.	[273]
	**SkQ1**	Penetrates the mitochondrial membrane to reduce oxidative damage and delay cell apoptosis.	Unique mitochondrial penetration mechanism for effective myocardial cell protection.	[274]
**Nrf2 Activators**	**Bardoxolone methyl**	Activates the Nrf2/ARE pathway to enhance antioxidant enzyme (e.g., HO-1) expression.	Enhances endogenous antioxidant capacity via transcriptional regulation, providing stable long-term effects.	[275]
**Small Molecule Scavengers**	**Edaravone derivatives**	Scavenges free radicals and reduces acute ROS levels.	Optimized molecular structure for higher efficiency and rapid action.	
	**Tempol**	Mimics superoxide dismutase (SOD) to inhibit superoxide production.	Simple synthetic pathway, providing an economical antioxidant treatment option.	
**Enzyme-Based Therapies**	**PEG-SOD/PEG-Catalase**	Modifies enzymes to extend circulation time and improve ROS scavenging capacity.	Enhanced stability and bioavailability, reducing the need for frequent administration.	
**ROS Metabolism Inhibitors**	**GKT137831**	Inhibits NOX to reduce ROS production.	High-specificity NOX inhibition with minimal side effects.	
	**p66Shc Inhibitor**	Targets upstream regulators of mitochondrial ROS production, delaying cardiovascular aging.	Innovative target directly addressing core mitochondrial oxidative stress mechanisms.	
**Multifunctional Antioxidant Molecules**	**RTA 408**	Simultaneously scavenges ROS and inhibits inflammation.	Integrated mechanism combining anti-inflammatory and antioxidant functions, broadening indications.	

### 4.2. Gene Therapy

Gene therapy, which has shown significant success in oncology and genetic disorders, is now being investigated for cardiovascular applications (Table 4). By modifying gene expression, this approach provides new therapeutic possibilities for conditions such as peripheral arterial disease and myocardial ischemia (Table 4) [276].

**Table 4:** Gene therapy for CVDs.

Typology	Advantages	Disadvantages	Related Research	Reference
**Gene editing**	High-precision targeted gene editing enabling permanent repair and reducing recurrence risk in monogenic diseases.	The potential risks of this technology include off-target mutations, immune responses, and challenges in precise delivery, which are coupled with high technical complexity and cost.	CRISPR-mediated gene editing ameliorates cardiac hypertrophy and improves cardiac function in a murine model of MYBPC3-related cardiomyopathy.	[277]
**Gene replacement therapy**	The precise targeting and repair of defective genes, leading to the restoration of normal cellular physiology, highlighting its broad clinical potential.	The limitations of this technology, including low insertion efficiency, off-target effects, and immunogenicity, necessitate further investigations into its long-term safety profile.	DMD gene replacement significantly improves cardiac function in a mouse model of Duchenne muscular dystrophy.	[278]
**RNA interference**	Highly specific targeting of specific gene expression to inhibit pathological effects of disease-causing genes; applicable to reversible regulation without affecting gene ontology; and short duration of action to modulate efficacy and safety.	Shorter expression time, requiring repeated administration; lower stability in vivo, difficult to resist enzymatic breakdown; complex drug delivery and dose regulation, frequent dosing cycles; and may trigger cytotoxic and immune responses.	RNAi inhibition of PCSK9 significantly reduces LDL-C levels and inhibits the course of atherosclerosis in *ApoE^−/−^* mice.	[279]
**Gene enhancement therapy**	Can upregulate protective gene expression and enhance antioxidant, anti-inflammatory and metabolic regulation functions; suitable for chronic diseases lacking protective mechanisms; and can delay lesions by modulating multiple protective pathways.	Higher dosage requirements, overexpression may trigger toxic effects; dependence on the level of gene expression makes it difficult to control the side effects of gene overexpression; the immune system may reject exogenous genes; and long-term monitoring and observation of safety is required.	SIRT1 overexpression protects against myocardial ischemia-reperfusion injury.	[280]
**AAV-mediated gene delivery**	Persistent gene expression, which facilitates long-term treatment; low immunogenicity, which reduces the risk of immune rejection; cardiac-specific delivery potential, which enhances therapeutic efficacy; and suitability for long-acting treatments for chronic diseases and genetic defects.	The limited capacity of AAV vectors restricts large-scale gene delivery; it is difficult to completely avoid immune recognition, so we need to be vigilant about potential immune reactions; the uneven integration of vectors may trigger gene insertion mutations; and the risk of long-term expression needs to be adequately verified.	AAV delivery of SERCA2a restores calcium homeostasis, reduces myocardial fibrosis, and restores cardiac function in mouse and porcine models of heart failure.	[281]
**Genetic vaccine**	Stimulates immune system regulation and reduces arterial inflammation and lipid accumulation; intervenes well in the early stages of disease; is easy to administer multiple times at relatively low cost; and can be personalised to improve vaccine specificity.	The immune response is difficult to control accurately and may trigger an autoimmune response; there are individual differences in vaccine tolerance and durability; the vaccine development and evaluation cycle is long; and some of the vaccine components may cause toxicity or side effects.	Gene vaccine targeting; CD68 and LDLR reduce arterial plaque formation and enhance anti-inflammation and lipid metabolism in a mouse model of atherosclerosis.	[282]

### 4.3. Cellular and Regenerative Medicine

#### 4.3.1. Cardiac Cell Therapy

Cardiac cell therapy is an emerging therapeutic strategy for heart diseases that involves transplanting healthy cells to repair damaged cardiac tissue (Table 4). These cells, which are derived from adult tissues such as bone marrow, skeletal muscle, the heart itself, or pluripotent stem cells, can function in two ways: (1) directly replacing damaged cardiac tissue to restore heart function and (2) indirectly promoting cardiac repair by secreting molecules and microvesicles that activate immune regulation and cardiac regenerative mechanisms [283]. Current regenerative approaches for CVDs primarily involve mesenchymal stem cells (MSCs), cancer stem cells (CSCs), induced pluripotent stem cells (iPSCs), and gene therapy [284]. MSCs, known for their safety and accessibility, are the most commonly employed [285]. However, their therapeutic effects are primarily mediated through paracrine mechanisms. While CSCs and iPSCs hold potential, they face challenges related to safety and technical feasibility.

Stem cell therapy holds immense promise for addressing oxidative stress in cardiovascular diseases through multifaceted mechanisms [286]. These include the secretion of antioxidative enzymes like superoxide dismutase (SOD) and glutathione peroxidase (GPx), effectively mitigating oxidative damage [287]. Furthermore, the release of vascular endothelial growth factor (VEGF) enhances angiogenesis, improving myocardial oxygenation and metabolism. Stem cells also modulate immune responses to alleviate inflammation while promoting myocardial regeneration via differentiation and paracrine signaling. Recent advancements in stem cell-derived exosome therapy offer targeted delivery of antioxidative molecules, reducing systemic side effects [288]. Integration with gene editing and nanotechnology further optimizes therapeutic efficacy, paving the way for transformative clinical applications.

#### 4.3.2. Exocrine Therapy

Exosome therapy is an innovative approach for treating CVDs that uses extracellular vesicles, known as exosomes, as therapeutic carriers [289]. Exosomes have gained recognition as versatile agents in cardiovascular health (Table 4). Studies have demonstrated that exosomes derived from various sources, including healthy individuals and cardiac surgery patients, can promote angiogenesis and protect cardiomyocytes [290]. Furthermore, animal models have shown that exosomes are actively released by cardiomyocytes and endothelial cells in response to ischemic injury, indicating their role in tissue repair and remodeling [291]. These findings highlight the potential of exosomes as promising therapeutic agents for CVDs.

Exosomal molecular markers, such as proteins, microRNAs (miRNAs), long non-coding RNAs (lncRNAs), and circulating DNA, provide valuable support for the early diagnosis of CVDs. Protein markers, including cardiac troponins (cTnI and cTnT), creatine kinase-MB (CK-MB), and inflammatory markers like tumor necrosis factor-alpha (TNF-α) and interleukin-6 (IL-6), can indicate early myocardial injury and inflammation [292]. MicroRNA markers such as *miR-21*, *miR-126*, *miR-1*, and *miR-133a* show early signs under conditions such as myocardial hypertrophy, endothelial cell repair, and acute myocardial infarction [293]. Long non-coding RNA (lncRNA) markers like *LIPCAR*, *H19*, *ANRIL*, and *KCNQ1OT1* are linked to myocardial remodeling, fibrosis, and plaque formation, suggesting long-term risks [294]. Additionally, increases in circulating DNA, including mitochondrial DNA (mtDNA) and cell-free DNA (cfDNA), can reflect cellular damage in acute events [289]. The combined detection of multiple molecular markers holds significant clinical value for the early diagnosis of CVDs.

Exosome therapy represents a promising emerging strategy for mitigating oxidative stress in cardiovascular diseases [111]. It functions primarily by delivering antioxidative enzymes, regulatory miRNAs (e.g., *miR-126* and *miR-210*), and proteins to reduce ROS accumulation [295]. Additionally, it modulates inflammatory pathways, breaking the vicious cycle between inflammation and oxidative stress while promoting angiogenesis and myocardial regeneration through the release of growth factors like VEGF and HGF [296]. By improving mitochondrial function and optimizing myocardial energy metabolism, exosome therapy demonstrates high specificity and minimal side effects. Integrated with gene editing and nanotechnology, it holds transformative potential for cardiovascular precision medicine.

### 4.4. Inflammation Regulation and Immunotherapy

IL-1β, a pro-inflammatory cytokine, plays a crucial role in the development of CVDs [297]. By activating signaling pathways such as NF-κB, IL-1β promotes inflammation and contributes to the formation and rupture of atherosclerotic plaques (Table 4). Canakinumab, a selective IL-1β receptor antagonist, has been shown to effectively inhibit inflammation and stabilize plaques, thereby reducing the risk of cardiovascular events [298]. Large-scale clinical trials have provided compelling evidence supporting the therapeutic efficacy of canakinumab in high-risk patients [299].

Anti-PCSK9 monoclonal antibodies have demonstrated significant potential in the treatment of CVDs [3]. By specifically binding to and inhibiting the PCSK9 protein, these antibodies upregulate the expression of LDL receptors (LDLR), leading to increased LDL clearance and reduced plasma LDL cholesterol (LDL-C) levels [300]. Large-scale randomized controlled trials have consistently shown that anti-PCSK9 antibody treatment significantly reduces LDL-C levels and the risk of major adverse cardiovascular events in high-risk patients [301,302]. These findings establish anti-PCSK9 antibodies as a valuable therapeutic option for managing CVDs.

### 4.5. Metabolic Regulator

Glucagon-like peptide-1 receptor agonists (GLP-1 RAs) have emerged as a novel class of anti-diabetic drugs with effects extending beyond glycemic control [303]. By mimicking the actions of endogenous glucagon-like peptide-1, GLP-1 RAs exert multifaceted effects on glucose and lipid metabolism. On the one hand, GLP-1 RAs promote pancreatic β-cell proliferation and function, enhance insulin secretion, and slow gastric emptying, leading to increased satiety and effective glycemic control [304]. On the other hand, GLP-1 RAs improve endothelial function, inhibit inflammation, and stabilize atherosclerotic plaques, thereby providing significant cardiovascular protection (Table 4). Compared with traditional anti-diabetic drugs, GLP-1 RAs have been shown to more effectively reduce the incidence of cardiovascular events, including nonfatal myocardial infarction, nonfatal stroke, and cardiovascular death, in patients with type 2 diabetes [305]. These findings suggest that GLP-1 RAs are not only effective anti-diabetic agents but also promising cardiovascular protective agents with broad clinical applications.

Sodium-glucose cotransporter 2 (SGLT2) inhibitors represent another class of novel anti-diabetic agents that have demonstrated remarkable efficacy in improving outcomes in patients with CVD [306]. By inhibiting the sodium-glucose cotransporter 2 in the proximal renal tubule, SGLT2 inhibitors effectively lower blood glucose levels and body weight, increase urinary sodium excretion, and reduce cardiac workload [307]. Additionally, SGLT2 inhibitors exert multiple beneficial effects, including improved myocardial energy metabolism, reduced myocardial hypertrophy, and attenuated inflammatory responses [308].

## 5. Outlook and Future Perspective

The evolving understanding of oxidative stress in CVD has significantly influenced current therapeutic strategies. Despite advances in identifying redox signaling pathways and their contributions to disease progression, challenges persist in translating these insights into effective, targeted therapies. Future research must continue to elucidate the complex interplay between oxidative stress and cardiovascular pathophysiology, particularly its stage-specific roles—from early endothelial injury to advanced cardiac remodeling. Characterizing these processes at various disease stages may help identify novel, stage-specific targets to enhance therapeutic precision.

Antioxidant therapies have shown promising potential, yet their clinical efficacy remains inconsistent, partly due to the nonspecific nature of many antioxidants and the complexities of redox signaling in diverse cellular contexts. To address these challenges, future studies should focus on developing selective antioxidants that target specific signaling pathways, such as Nrf2/Keap1/ARE, SIRT1, and PI3K/Akt, while avoiding interference with essential physiological redox processes. Further exploration of cross-talk between these pathways may reveal synergies that could be leveraged in multi-target therapies for more robust cardiovascular protection.

Emerging strategies, including gene therapy, cellular and regenerative approaches, and inflammation regulation, offer a promising future for the treatment of CVDs driven by oxidative stress. Gene therapy holds particular promise in addressing genetic predispositions and modifying redox-sensitive genes to enhance antioxidant capacity. Meanwhile, cell-based therapies and regenerative medicine may facilitate tissue repair and functional recovery after injury. Additionally, immunomodulatory approaches that mitigate inflammation could complement antioxidant strategies, offering dual benefits in redox and immune regulation.

Ultimately, integrating metabolic regulation with oxidative stress management represents a promising frontier. Modulating metabolic pathways, including those involving AMPK and mitochondrial dynamics, may reduce oxidative stress and enhance cellular resilience, offering a holistic approach to CVD management. For successful clinical translation, future investigations must prioritize refining these novel interventions within robust preclinical and clinical frameworks, emphasizing safety, efficacy, and precision targeting. Such multifaceted approaches are likely to pave the way for more effective and personalized cardiovascular therapies, ultimately reducing the global burden of CVD.
